# Imbalance in glycemic control between the treatment and placebo groups in cardiovascular outcome trials in type 2 diabetes

**DOI:** 10.1186/s40545-019-0193-y

**Published:** 2019-11-18

**Authors:** Rumiko Shimazawa, Masayuki Ikeda

**Affiliations:** 1 Department of Clinical Pharmacology, Tokai University School of Medicine, Isehara, Kanagawa 259-1193 Japan; 2 Department of Medical Informatics, Kagawa University Hospital, Miki-cho, Kagawa 761-0793 Japan

**Keywords:** Bias, Cardiovascular diseases, Clinical trials, Drug approval, Glycated hemoglobin, Hypoglycemic agents, Type 2 diabetes mellitus

## Abstract

**Background:**

Glycated hemoglobin (HbA1c) is accepted as the most reliable marker for assessing chronic glycemia. The present study aimed to investigate glycemic control in cardiovascular outcome trials (CVOTs) performed by pharmaceutical sponsors, at the request of the United States Food and Drug Administration (FDA) to ensure that newer hypoglycemic agents do not increase cardiovascular risk for patients with type 2 diabetes.

**Methods:**

We chose ClinicalTrials.gov as a data source to identify randomized, double-blind, placebo-controlled non-inferiority trials of newer hypoglycemic agents for which the FDA 2008 guidance required a CVOT involving patients with type 2 diabetes.

**Results:**

We identified 12 CVOTs, all of which were performed in accordance with the FDA guidance and published as of December 2018. Participants received either active treatment or placebo in addition to their existing therapy. On the assumption that HbA1c concentrations would be higher in the placebo group than in the treatment group, the use of open-label glucose lowering agents was encouraged as required to help all patients reach appropriate HbA1c targets according to local guidelines. As a result, the number of patients who received additional hypoglycemic agents during the trial was greater in the placebo group than in the treatment group in 10 of the CVOTs. Although the CVOTs were designed to avoid any imbalance in glycemic control between the groups, HbA1c concentrations were substantially higher in the placebo group than in the treatment group in all CVOTs throughout the observational period. The inferior glycemic control in the placebo groups was not considered in analyzing the outcomes in any of the CVOTs.

**Conclusions:**

The safety and efficacy of new hypoglycemic agents are potentially inflated because the participants in the placebo groups unexpectedly exhibited inferior glycemic control throughout the trial compared with the outcomes in the treatment groups. This imbalance may distort data interpretation and mask potential risks of the drugs. Re-analysis with adjustment for HbA1c concentrations would determine whether the results of these CVOTs were biased by the difference in glycemic control between the treatment and placebo groups and reveal potential effects of the test drugs independent of glycemic control.

## Background

Diabetes treatment is adjusted on the basis of the concentrations of glycated hemoglobin (HbA1c), which is accepted as the most reliable marker to assess chromic glycemia [1]. Regulatory authorities have approved hypoglycemic agents using HbA1c concentrations as the outcome because good glycemic control (defined by specific HbA1c targets) is expected to reduce cardiovascular (CV) risk in patients with type 2 diabetes mellitus (T2D) [2, 3].

This glucocentric approach, however, has been challenged because of controversy over the CV risk associated with specific hypoglycemic agents, notably the peroxisome proliferator-activated receptor agonists, muraglitazar and rosiglitazone [4]. Compared with placebo or pioglitazone, muraglitazar was associated with a greater than 2-fold (relative risk = 2.62, 95% confidence interval [CI] = 1.36–5.05; *P* = 0.004) higher incidence of CV events, including all-cause mortality, myocardial infarction, heart failure, and stroke [5]. This report prompted Bristol-Meyers Squibb to withdraw its new drug application for muraglitazar even though the drug had received a recommendation for approval from a United States Food and Drug Administration (FDA) advisory panel [6]. A meta-analysis of rosiglitazone [7] illustrated that the drug was associated with a significant increase in the risk of myocardial infarction (odds ratio = 1.43, 95% CI = 1.03–1.98; *P* = 0.03). In a trial evaluating CV outcomes in rosiglitazone-treated patients with T2D [8], hospital admission for heart failure or death was more frequent in the rosiglitazone group than in the active control group (hazard ratio [HR] = 2.10, 95% CI = 1.35–3.27; *P* = 0.001).

These data prompted the FDA to ask sponsors to perform trials as a post-marketing requirement to ensure that new hypoglycemic agents do not increase CV risk to an unacceptable extent. Since the FDA published the trial guidance in December 2008 [9], the number of CV outcome trials (CVOTs) in T2D [10, 11] has greatly increased [12]. The FDA guidance states that reliance on HbA1c concentrations remain an acceptable primary efficacy endpoint for approving drugs for the treatment of hyperglycemia secondary to diabetes.

Although the CVOTs were designed to achieve comparable glycemic control between the trial arms, the patients in the placebo group were reported to have higher HbA1c concentrations than those in the test drug group in some of the trials [10, 13]. This difference in glycemic control has the potential to distort data interpretation and mask the risks of the tested hypoglycemic agents. However, this issue has never been studied in detail; instead, it has been dismissed as negligible on the assumption that HbA1c is not the right outcome for studies of diabetes [14, 15], even though this assumption remains to be validated. Taking these concerns about HbA1c into account, we investigated the differences in glycemic control in the CVOTs performed according the FDA 2008 guidance [9] and evaluated the effects of these differences on the safety and efficacy of the tested hypoglycemic agents.

## Methods

To determine the target CVOTs, we identified newer FDA-approved hypoglycemic agents [16], i.e., dipeptidyl peptidase-4 (DPP-4) inhibitors, glucagon-like peptide 1 (GLP-1) agonists, and sodium glucose cotransporter 2 (SGLT2) inhibitors, indicated for T2D, focusing on studies for which CVOTs were required by the agency [4]. Then, we retrieved CVOTs for these newer hypoglycemic agents designed according to the FDA 2008 guidance [9] and from ClinicalTrials.gov [17], which was a sufficient source for such studies because any trial compliant to the guidance [9] was subject to registration on ClinicalTrials.gov [18]. The selection criteria for CVOTs other than compliance with FDA guidance [9] were as follows: randomized, double-blind, placebo-controlled trial including patients with T2D, and the primary endpoint was “Major Cardiac Adverse Events (MACE).”

## Results

### Participant characteristics and trial design (Table 1)

We identified 12 CVOTs [19–32], all of which were performed in accordance with the FDA guidance [9] and published as of December 31, 2018. The test drugs were DPP-4 inhibitors (*n* = 4) [19–23], GLP-1 agonists (*n* = 5) [24–28], and SGLT2 inhibitors (*n* = 3) [29–32]. All of the CVOTs were sponsored by the pharmaceutical companies that had developed the test drugs. Participants had advanced atherosclerotic CV risk or established CV diseases. The patients had a long duration of T2D (mean, 7.1–15.0 years). The baseline mean HbA1c concentrations ranged from 7.2 to 8.7%. The participants received either active treatment or placebo in addition to their existing therapy. In other words, the comparator arm was not a true placebo because additional glucose-lowering therapies were allowed, even though each of the trials was described as being placebo-controlled. These CVOTs were primarily designed to rule out unacceptable CV risk, but some were powered to reveal superiority after non-inferiority was demonstrated. The primary outcome was four-component MACE (CV death, nonfatal myocardial infarction, nonfatal stroke, and hospitalization for unstable angina) in two trials [23, 27], whereas, the primary endpoint was three-component MACE (CV death, nonfatal myocardial infarction, and nonfatal stroke) in the other 10 trials.

**Table 1:** Baseline characteristics of the trial participants and design features of the cardiovascular outcome trials

Trial	Drug (Class)	Year started/reported	Number of subjects	Age (years)	Baseline HbA1c (%)	BMI	Diabetes duration (years)	Prior CVD/HF (%)	Follow up (median, years)	Primary endpoints
EXAMINE	Alogliptin (DPP-4)	2009/13	5380	61.0	8.0	28.7	7.1	100/28	1.5	3 MACE
CARMELINA	Linagliptin (DPP-4)	2013/18	6979	66.1	8.0	31.4	15.0	58/27	2.2	3 MACE
SAVOR-TIMI	Saxagliptin (DPP-4)	2010/13	16,492	65.1	8.0	31.1	10.3	78/13	2.1	3 MACE
TECOS	Sitagliptin (DPP-4)	2008/15	14,671	65.4	7.2	30.2	11.6	74/18	3.0	4 MACE
HARMONY	Albiglutide (GLP-1)	2015/18	9463	64.1	8.7	32.3	14.1	70/20	1.6	3 MACE
EXSCEL	Exenatide (GLP-1)	2010/17	14,752	62.0	8.0	31.8	12.0	73/16	3.2	3 MACE
LEADER	Liraglutide (GLP-1)	2010/16	9340	64.3	8.7	32.5	12.8	81/18	3.8	3 MACE
ELIXA	Lixisenatide (GLP-1)	2010/15	6068	60.3	7.7	30.2	9.3	100/22	2.1	4 MACE
SUSTAIN-6	Semaglutide (GLP-1)	2013/16	3297	64.6	8.7	32.8	13.9	60/24	2.1	3 MACE
CANVAS	Canagliflozin (SGLT2)	2009/17	10,142	63.3	8.2	32.0	13.5	66/14	3.6	3 MACE
DECLARE–TIMI	Dapagliflozin (SGLT2)	2013/18	17,160	63.9	8.3	32.1	11.0	41/10	4.2	3 MACE
EMPA-REG OUTCOME	Empagliflozin (SGLT2)	2010/15	7028	63.1	8.1	30.7	57% > 10 years	99/10	3.1	3 MACE

*BMI* Body mass index, *CVD* Cardiovascular disease, *DPP-4* Dipeptidyl peptidase-4 inhibitor, *GLP-1* Glucagon-like peptide 1 agonist, *HF* Heart failure, *3 MACE* Three-component major adverse cardiovascular events (cardiovascular death, nonfatal myocardial infarction, and nonfatal stroke), *4 MACE* Four-component major adverse cardiovascular events (cardiovascular death, nonfatal myocardial infarction, nonfatal stroke, and hospitalization for unstable angina), *SGLT2* Sodium glucose cotransporter 2 inhibitor

### Glucose control and adverse events in the CVOTs (Table 2)

On the assumption that HbA1c concentrations would be higher in the placebo group than in the treatment group, the use of open-label hypoglycemic agents was encouraged as required to help all patients reach appropriate HbA1c targets according to local guidelines. Other CV risk factors (e.g., blood pressure, lipids) were also managed on the basis of local guidelines. The sources of data for hypoglycemic agents at baseline and during each trial were provided as supplementary appendices in the original reports. The number of patients who received additional hypoglycemic agents during the trial was significantly greater in the placebo group than in the treatment group in 10 of the CVOTs, i.e., CARMELINA [21], SAVOR-TIMI [22], TECOS [23], HARMONY [24], EXSCEL [25], LEADER [26], SUSTAIN-6 [28], CANVAS [29], DECLARE–TIMI [30], and EMPA-REG OUTCOME [31, 32]. In the other two trials, namely the EXAMINE [19, 20] and ELIXA trials [27], hypoglycemic agents introduced post-baseline were not reported either in the article or its supplementary appendix. Regardless of whether a greater number of patients in the placebo group received additional hypoglycemic agents, HbA1c concentrations were substantially higher (from 0.27 to 1.00) in the placebo group than in the treatment group in all CVOTs throughout the observational period (second column from the left in Table 2). This difference in glycemic control was statistically significant in all CVOTs. In several trials, unfavorable CV events were more frequent in the treatment group (despite better glycemic control) than in the placebo group. In the EXAMINE trial [20], among participants without a history of heart failure at baseline, the risk of hospital admission for heart failure was significantly higher in the alogliptin group than in the placebo group. The SAVOR-TIMI trial [22] illustrated that more patients were hospitalized for heart failure in the saxagliptin group than in the placebo group. The CANVAS [29] trial found that canagliflozin doubled the risk for lower-limb amputation.

**Table 2:** HbA1c imbalance, additional hypoglycemic agents, and outcomes in the cardiovascular outcome trials

Trial	HbA1c imbalance^a^	Additional hypoglycemic agents	Primary endpoint	Cardiovascular death	Nonfatal MI	Nonfatal Stroke	Heart failure	Death from any cause	Increased adverse events^b^
EXAMINE	0.36 (mean)	NA	0.96 (0.8–1.16)	0.79 (0.60–1.04)	1.08 (0.88–1.33)	0.91 (0.55–1.50)	**1.76** ^c^ **(1.07–2.90)**	0.88 (0.71–1.13)	Heart failure
CARMELINA	0.36 (mean)	More in P group	1.02 (0.89–1.17)	0.96 (0.81–1.14)	1.15 (0.91–1.45)	0.88 (0.63–1.23)	0.90 (0.74–1.08)	0.98 (0.84–1.09)	
SAVOR-TIMI	0.3 (52^d^)	More in P group	1.00 (0.98–1.12)	1.03 (0.87–1.22)	0.95 (0.80–1.12)	1.11 (0.88–1.39)	**1.27 (1.07–1.51)**	1.11 (0.96–1.27)	Heart failure
TECOS	0.29 (mean)	More in P group	0.98 (0.89–1.08)	1.03 (0.89–1.19)	0.96 (0.81–1.13)^e^	0.93 (0.75–1.16) ^f^	1.00 (0.83–1.20)	1.01 (0.90–1.14)	
HARMONY	0.63 (8^d^)	More in P group	*0.78 (0.68*–*0.90)*	0.93 (0.73–1.19)	*0.75 (0.61–0.90)* ^e^	0.86 (0.66–1.14) ^f^	0.85 (0.70–1.04)	0.95 (0.79–1.16)	
EXSCEL	0.53 (mean)	More in P group	0.91 (0.83–1.00)	0.88 (0.73–1.05)	0.95 (0.84–1.09)	0.86 (0.70–1.07)	0.94 (0.78–1.13)	*0.86 (0.77–0.97)*	
LEADER	0.4 (36^d^)	More in P group	*0.87 (0.78–0.97)*	*0.78 (0.66–0.93)*	0.88 (0.75–1.03)	0.89 (0.72–1.11)	0.87 (0.73–1.05)	*0.85 (0.74–0.97)*	
ELIXA	0.27 (mean)	NA	1.02 (0.89–1.17)	0.98 (0.78–1.22)	1.03 (0.87–1.22)	1.12 (0.79–1.58)	0.96 (0.75–1.23)	0.94 (0.78–1.13)	
SUSTAIN-6	1.00 (104^d^)	More in P group	*0.74 (0.58–0.95)*	0.98 (0.65–1.48)	0.74 (0.51–1.08)	*0.61 (0.38–0.99)*	1.11 (0.77–1.61)	1.05 (0.74–1.50)	
CANVAS	0.58 (mean)	More in P group	*0.86 (0.75–0.97)*	0.87 (0.72–1.06)	0.85 (0.69–1.05)	0.90 (0.71–1.15)	*0.67 (0.52–0.87)*	0.87 (0.74–1.01)	Amputation
DECLARE–TIMI	0.42 (mean)	More in P group	0.93 (0.84–1.03)	0.98 (0.82–1.17)	0.89 (0.77–1.01)	1.01 (0.84–1.21)	*0.73 (0.61–0.88)*	0.93 (0.82–1.04)	Ketoacidosis Genital infection
EMPA-REG OUTCOME	0.47 (94^d^)	More in P group	*0.86 (0.74–0.99)*	*0.62 (0.49–0.77)*	0.87 (0.70–1.09)	1.24 (0.92–1.67)	*0.65 (0.50–0.85)*	*0.68 (0.57–0.82)*	

^a^HbA1c concentrations were significantly higher in the placebo group than in the treatment group. ^b^Adverse events that had a significant increase in frequency in the treatment group. ^c^In patients without a history of heart failure at baseline.^d^Week at which the data were obtained. ^e^Fatal myocardial infarction included. ^f^Fatal stroke included. Data are presented as the hazard ratio (95% confidence interval). Bold type represents a significant increase of the event in the treatment group compared with that in the placebo group. Italic type represents a significant reduction. *MI* Myocardial infarction, *NA* Data not available, *P* Placebo

### Macrovascular outcomes (Table 2)

In five trials (HARMONY [24], LEADER [26], SUSTAIN-6 [28], CANVAS [29], and EMPA-REG OUTCOME [31, 32]), the primary outcome was achieved in significantly fewer patients in the treatment group than in the placebo group, whereas no significant difference in the primary outcome was observed between the treatment and placebo groups in the other seven trials. Among the CV events, the event for which a significant reduction in risk was observed in the treatment group differed depending upon the trials as follows: CV death was reduced in the LEADER [26] and EMPA-REG OUTCOME [31, 32] trials; hospital admission for heart failure was reduced in the CANVAS [29], DECLARE–TIMI [30], and EMPA-REG OUTCOME [31, 32] trials; myocardial infarction was reduced only in the HARMONY [24] trial; nonfatal stroke was reduced only in the SUSTAIN-6 [28] trial; and heart failure was reduced in the CANVAS [29], DECLARE–TIMI [30], and EMPA-REG OUTCOME [31, 32] trials.

### Microvascular outcomes

Although nephropathy was evaluated in all CVOTs, the outcome measure varied and the effects were inconsistent. The estimated glomerular filtration rate was comparable between the treatment and placebo groups in the EXAMINE [19, 20] and HARMONY [24] trials, whereas it was lower in the sitagliptin group in the TECOS trial [23]. The urinary albumin-to-creatinine ratio (UACR) showed significantly less worsening in the linagliptin and saxagliptin groups in the CARMELINA [21] and SAVOR-TIMI [22] trials, respectively. In the ELIXA [27] trial, the pre-specified analysis of the percentage change in the UACR from baseline to 108 weeks was significantly different in favor of lixisenatide over placebo (24% vs. 34%; *P* = 0.004), but upon post hoc analysis with adjustment for HbA1c, this difference was attenuated (*P* = 0.07) [27]. In the EXSCEL [25] trial, no significant difference was observed in the incidence of micro- and macroalbuminuria between the exenatide and placebo groups. In the LEADER [26], SUSTAIN-6 [28], CANVAS [29], DECLARE–TIMI [30], and EMPA-REG OUTCOME [31, 32] trials, the renal composite endpoint was significantly reduced in in favor of the treatment group. Retinopathy, which was included in as an outcome measure in five of the CVOTs, had a comparable frequency between the groups in EXSCEL [25], whereas it was more frequent in the treatment group in the TECOS [23], LEADER [26], and SUSTAIN-6 [28] trials. In the CARMELINA [21] trial, retinopathy was less common in the linagliptin group. Neuropathy, which was evaluated only in the EXSCEL [25] trial, did not have a significantly different incidence between the groups.

## Discussion

These CVOTs generated interpretative challenges. The use of open-label hypoglycemic agents was encouraged to minimize the confounding effects of differences in glycemic control. Despite the cautious designs, HbA1c concentrations were substantially higher in the placebo group than in the treatment group in all of the CVOTs. This imbalance, which can potentially obscure the risk of the test drug, means that subjects in the placebo group had inferior glycemic control than those in the treatment group. Of note, the investigators of the EXSCEL [25] trial stated that the significant reduction in the risk of death from any cause in the exenatide group might have been influenced by the inferior glycemic control in the placebo group. The non-glucose effects that are often associated with newer hypoglycemic agents [12], e.g., changes in body weight, blood pressure, and LDL-cholesterol, could also introduce imbalances. These confounders challenge the interpretation of outcomes for the hypoglycemic agents tested in the CVOTs. The greater use of additional hypoglycemic agents in the placebo group than the treatment group in 10 of the CVOTs represented another potential confounder. The imbalance of HbA1c concentrations between 0.27 and 1.00% should not be dismissed because it is comparable to that in the UKPDS 34 study [3], in which the median HbA1c concentrations during 10 years of follow-up were 7.4% in the metformin group and 8.0% in the conventional treatment group. Adjustment for predictive baseline characteristics, even when largely balanced, may lead to clearly different estimates of the effect of treatment on CV outcomes [33]. Re-analysis of the data in the CVOTs with adjustment for HbA1c would determine whether the results were biased by the difference in glycemic control between the treatment and placebo groups and reveal the potential effects of the test drugs independent of glycemic control [34, 35].

The primary endpoint that was used in all of the CVOTs complicated the evaluation of efficacy. The use of composite endpoints [36] can potentially distort trials of a new antidiabetic therapies for treating T2D. In particular, whereas the CVOTs aimed to establish safety, composite endpoints were originally designed to determine overall efficacy. We cannot determine whether any of the observed difference between the treatment and placebo arms are independent of glycemic control. Although the imbalance was present in all of the CVOTs, none provided data corrected for the imbalance in HbA1c. Only the post hoc analysis of the EXAMINE trial [37] revealed the absence of relationships of baseline HbA1c or HbA1c after 1 month of treatment with the risk of MACE. To draw a more robust conclusion, further in-depth analyses considering the aforementioned biases, including the imbalance in HbA1c between the treatment and placebo groups, are required.

Glycemic control is expected to reduce the risk of heart failure [38], which is generally regarded as a CV event. However, only three of the CVOTs (the CANVAS [29], DECLARE–TIMI [30], and EMPA-REG OUTCOME [31, 32] trials) reported reductions in heart failure risk despite the substantially lower HbA1c concentrations in the treatment group than in the placebo group in all of the CVOTs. Indeed, in the EXAMINE [19, 20] and SAVOR-TIMI [22] trials, the risk of heart failure was higher in the treatment group than in the placebo group. In response to these results, the FDA added a heart failure warning to the labels of alogliptin and saxagliptin [39]. The agency subsequently added the same warning to the labels of linagliptin [40] and sitagliptin [41] despite no finding of an increase in heart failure risk in either the CARMELINA [21] or TECOS [23] trial, and the association between DPP-4 inhibitors and heart failure as a class effect remains uncertain [42].

Whereas GLP-1 agonists reduced the risk of myocardial ischemia and heart failure in the early studies [43, 44], no reduction in heart failure risk was observed in the HARMONY [24], EXSCEL [25], LEADER [26], ELIXA [27], or SUSTAIN-6 [28] trials despite the substantially lower HbA1c concentrations in the treatment group than in the placebo group. Liraglutide was studied in patients with T2D and heart failure in two smaller trials in addition to the five CVOTs of GLP-1 agonists reviewed in this study. In the LIVE trial [45], whereas 24 weeks of liraglutide treatment had no effect on left ventricular systolic function in 241 patients with T2D and chronic heart failure, a higher rate of adverse cardiac events was observed (12 in the liraglutide group versus three in the placebo group). The FIGHT trial [46], which enrolled 178 patients with T2D and 122 without T2D to test whether liraglutide improves clinical stability following hospitalization for acute heart failure, failed to demonstrate any benefit of liraglutide. Moreover, subgroup analysis of patients with T2D revealed a non-significant increase in the number of deaths or re-hospitalizations for heart failure (HR = 1.54, 95% CI = 0.97–2.46; *P* = 0.07) in the liraglutide group compared with the placebo group. On the basis of the fact that only 18% of the LEADER [26] trial population was reported to have heart failure at baseline, the results of the LIVE and FIGHT trials raise safety concerns regarding the use of liraglutide in patients with T2D and heart failure.

In contrast to DPP-4 inhibitors and GLP-1 agonists, the SGLT2 inhibitors canagliflozin, dapagliflozin, and empagliflozin reduced the risk of heart failure in the CANVAS [29], DECLARE–TIMI [30], and EMPA-REG OUTCOME [31, 32] trials, respectively. However, we should exercise caution in interpreting these data because the inferior glycemic control in the placebo group might have affected the outcome in favor of the treatment group. These SGLT2 inhibitors were reported to be associated with a 2-fold higher risk for amputation in a register-based cohort study [47]. This may be a class effect of SGLT2 inhibitors rather than an effect specific to canagliflozin (which has a boxed warning about amputation on its label) because the proportions of patients who used dapagliflozin, empagliflozin, and canagliflozin in the study were 61, 38, and 1%, respectively. Regarding microvascular outcomes, the attenuation of the favorable UACR outcome for lixisenatide after adjustment for HbA1c [27] indicates a need for comprehensive re-analysis of the data in the CVOTs. The increase in the risk of retinopathy observed in the TECOS [23], LEADER [26], and SUSTAIN-6 [28] trials represents another concern.

Our study found that lower HbA1c concentrations in the treatment group throughout the trial do not necessarily improve CV outcomes in patients with T2D. HbA1c remains the best marker for monitoring drug effects on T2D, and the strengths and limitations of HbA1c first need to be determined. Biological variation in hemoglobin glycation [48] is one of the new research directions to validate HbA1c as a surrogate in T2D [49]. Monitoring blood glucose and HbA1c concentrations closely in randomized controlled trials may therefore be a promising option to assess the biological variation in HbA1c.

Our study has limitations. First, its cross-sectional study design does not permit determinations of cause and effect, making interpretation of the data inferential. We could not identify the cause of the imbalance of glycemic control between the treatment and placebo groups or discriminate any confounding effects. Second, we did not have the data for re-analysis to investigate the effects of the imbalance of glycemic control on the results of the CVOTs, which must be a focus of public attention. These limitations restrict the applicability of our study results to other studies.

## Conclusion

The safety and efficacy of new hypoglycemic agents are of particularly great concern to patients with diabetes. The imbalance in glycemic control observed in CVOTs evaluating hypoglycemic drugs placed the patients in the placebo groups at potential risk for CV events. Misinterpretation of data from these trials could lead to incorrect evaluation of both the efficacy and safety of the drug and eventually harm patients. Our study will help the FDA and other regulatory bodies to critically review CVOTs in this area from a regulatory perspective because additional CVOTs to address the FDA 2008 guidance are ongoing. The FDA should require re-analysis with adjustment for HbA1c to rule out potential biases against the safety and efficacy of the tested drug, which are of crucial concern. The re-analysis would determine whether the results of the CVOTs were biased by the difference in glycemic control between the treatment and placebo groups. Further review of these CVOTs regarding the imbalance of HbA1c will lead to precise interpretation of the data and correct evaluation of the tested hypoglycemic agents in future trials.

## Data Availability

All data generated or analyzed during this study are included in this published article.

## Abbreviations

CANVAS: Canagliflozin Cardiovascular Assessment Study
CARMELINA: Cardiovascular and Renal Microvascular Outcome Study With Linagliptin
CI: Confidence interval
CV: Cardiovascular
CVOTs: Cardiovascular outcome trials
DECLARE–TIMI: Dapagliflozin Effect on Cardiovascular Events–Thrombolysis in Myocardial Infarction
DPP-4: Dipeptidyl peptidase-4
ELIXA: Evaluation of Lixisenatide in Acute Coronary Syndrome
EMPA-REG OUTCOME: Empagliflozin, Cardiovascular Outcomes, and Mortality in Type 2 Diabetes
EXAMINE: Examination of Cardiovascular Outcomes with Alogliptin versus Standard of Care
EXSCEL: Exenatide Study of Cardiovascular Event Lowering
FDA: United States Food and Drug Administration
FIGHT: Functional Impact of GLP-1 for Heart Failure Treatment
GLP-1: glucagon-like peptide-1
HARMONY: Albiglutide and cardiovascular outcomes in patients with type 2 diabetes and cardiovascular disease
HbA1c: Glycated hemoglobin
HR: hazard ratio
LEADER: Liraglutide Effect and Action in Diabetes Evaluation of Cardiovascular Outcome Results
LIVE: Effect of Liraglutide on Left Ventricular Function in Chronic Heart Failure Patients With and Without Type 2 Diabetes
MACE: major adverse cardiovascular events
SAVOR-TIMI: Saxagliptin Assessment of Vascular Outcomes Recorded in Patients with Diabetes Mellitus–Thrombolysis in Myocardial Infarction
SGLT2: sodium glucose cotransporter 2
SUSTAIN 6: Semaglutide and Cardiovascular Outcomes in Patients with Type 2 Diabetes
T2D: Type 2 diabetes mellitus
TECOS: Trial Evaluating Cardiovascular Outcomes with Sitagliptin
UACR: Urinary albumin to creatinine ratio
UKPDS: United Kingdom Prospective Diabetes Study
